# Clinical and genetic features of a cohort of patients with *MFN2*-related neuropathy

**DOI:** 10.1038/s41598-022-10220-0

**Published:** 2022-04-13

**Authors:** Elena Abati, Arianna Manini, Daniele Velardo, Roberto Del Bo, Laura Napoli, Federica Rizzo, Maurizio Moggio, Nereo Bresolin, Emilia Bellone, Maria Teresa Bassi, Maria Grazia D’Angelo, Giacomo Pietro Comi, Stefania Corti

**Affiliations:** 1 Department of Pathophysiology and Transplantation (DEPT), Dino Ferrari Centre, Neuroscience Section, Fondazione IRCCS Ca’ Granda - Ospedale Maggiore Policlinico, University of Milan, Via Francesco Sforza 35, 20122 Milan, Italy; 2 Neurology Unit, Fondazione IRCCS Ca’ Granda Ospedale Maggiore Policlinico, Milan, Italy; 3 Neuromuscular and Rare Diseases Unit, Fondazione IRCCS Ca’ Granda Ospedale Maggiore Policlinico, Milan, Italy; 4 Department of Neuroscience, Rehabilitation, Ophthalmology, Genetics, Maternal and Child Health (Dinogmi) – Medical Genetics, University of Genoa, Genoa, Italy; 5 Laboratory of Molecular Biology, Scientific Institute IRCCS E. Medea, Bosisio Parini, Lecco, Italy; 6 Neuromuscular Disorder Unit, Scientific Institute IRCCS E. Medea, Bosisio Parini, Lecco, Italy

**Keywords:** Neurological disorders, Genetics research

## Abstract

Charcot–Marie–Tooth disease type 2A (CMT2A) is a rare inherited axonal neuropathy caused by mutations in *MFN2* gene, which encodes Mitofusin 2, a transmembrane protein of the outer mitochondrial membrane. We performed a cross-sectional analysis on thirteen patients carrying mutations in *MFN2*, from ten families, describing their clinical and genetic characteristics. Evaluated patients presented a variable age of onset and a wide phenotypic spectrum, with most patients presenting a severe phenotype. A novel heterozygous missense variant was detected, p.K357E. It is located at a highly conserved position and predicted as pathogenic by in silico tools. At a clinical level, the p.K357E carrier shows a severe sensorimotor axonal neuropathy. In conclusion, our work expands the genetic spectrum of CMT2A, disclosing a novel mutation and its related clinical effect, and provides a detailed description of the clinical features of a cohort of patients with *MFN2* mutations. Obtaining a precise genetic diagnosis in affected families is crucial both for family planning and prenatal diagnosis, and in a therapeutic perspective, as we are entering the era of personalized therapy for genetic diseases.

## Introduction

Charcot–Marie–Tooth disease (CMT) includes a wide spectrum of primary inherited sensory-motor neuropathies associated with more than 100 different genetic culprits^1^. With an overall prevalence of 1/1200-2500, it represents the most common genetically inherited neuromuscular disorder^2^. CMTs are classified according to their neurophysiological properties and inheritance pattern. Demyelinating CMT type 1 is characterized by reduced motor nerve conduction velocity (MNCV), while axonal CMT type 2 shows preserved MNCV^1^. Among CMT2, CMT2A is the most frequent form, accounting for approximately 10–40% of axonal CMT cases and 4–7% of all CMTs with a genetic diagnosis^2–6^.

**Figure 1:**
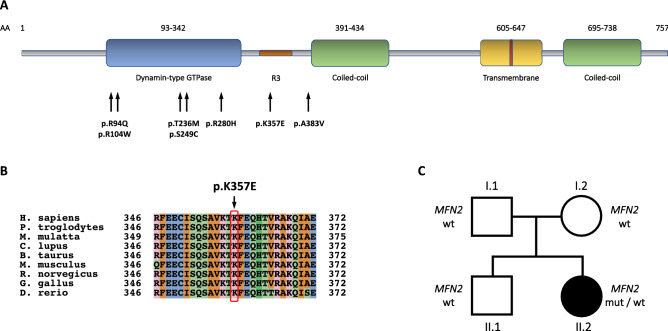
Genetic and bioinformatic analysis. (**A**) Schematic representation of MFN2 protein structure, showing the localization of all the mutations detected in the current cohort (**B**) Conservation among orthologous genes of the K357 amino acid (mutation site), with the colours used by the Clustal Omega multiple sequence alignment program (red = hydrophobic, light blue = positively charged, pink = negatively charged, light green = polar, aquamarine = aromatic, dark green = glycine, orange = proline)^49^ (**C**) Pedigree of the patient carrying the p.K357E *MFN2* variant. Circles correspond to females, squares to males. The black symbol indicates the proband affected by CMT2A, whereas the white ones are used for healthy relatives.

CMT2A is associated with mutations in the nuclear-encoded mitochondrial gene *mitofusin 2 (MFN2),* which is translated into the 757-amino acid long protein Mitofusin2 (MFN2). MFN2 is a highly conserved GTPase, anchored to the outer mitochondrial membrane via two adjacent transmembrane regions (Fig. 1A). Together with its homolog Mitofusin 1 (MFN1), it is implicated in the regulation of the balance between mitochondrial fusion and fission, two processes that are deemed critical in mitochondrial quality control, cellular stress response and apoptosis^7^.

More than 100 *MFN2* mutations have been detected in CMT2A patients; most of them are missense, whereas a limited number are nonsense variants or deletions. Although few recessive and semidominant forms have been described^8–11^, CMT2A is generally associated with autosomal dominant inheritance^5,12–16^. As described in other case series, most of mutations locate within the highly conserved GTPase or coiled-coil domains (Fig. 1A)^16^.

CMT2A has been associated to an early-onset, severe motor-predominant neuropathy, accompanied in some cases by significant proprioception loss^15,16^. In the largest cohort study of CMT2A natural history, childhood onset was shown to be the most predictive marker of disease severity^16^. Additional symptoms may be present and include optic nerve atrophy, scoliosis, hearing loss, vocal cord paralysis and upper motor neuron involvement^15–19^. White matter lesions can be detected on magnetic resonance imaging (MRI)^20,21^. Moreover, specific variants (e.g. those involving the arginine at the amino acid residue 104) have been associated with a neuropsychiatric syndrome characterized by developmental delay and cognitive impairment^22^. Overall, a wide inter-individual variability has been observed. Genotype–phenotype studies have been attempted, showing significant heterogeneity, also among members of the same family^15–18,23^.

Currently, no curative treatment is available for CMT2A, although MFN2 agonists and histone-deacetylase inhibitors have shown promising results in experimental models^24,25^. Furthermore, gene therapy-based approaches are currently under investigation and pledge to revolutionize the therapeutic options of rare diseases^26,27^. In this perspective, unraveling the genetic and phenotypic spectrum of CMT2A is becoming of utmost importance.

Our study analyzes thirteen patients from ten families, describing their clinical and genetic characteristics.

## Patients and methods

### Patients’ clinical evaluation

Between January 2018 and December 2020, we recruited 13 patients belonging to 10 CMT2A families with confirmed heterozygous *MFN2* mutations. Three patients from the same pedigree were already described by our group in a previously published work^22^; we report new clinical data. Neurological evaluation and chart reviews was performed by neurologists experienced in neuromuscular diseases of the IRCCS Fondazione Ca’ Granda Ospedale Maggiore Policlinico. CMT2A was defined as inherited axonal neuropathy with nerve conduction velocities in the upper extremity > 38 m/s associated with *MFN2* mutations. Personal and family history was investigated and common acquired causes of axonal neuropathy were excluded. Standard clinical information was obtained, including previous neurophysiologic studies, MRI and sural nerve or muscle biopsies. Muscle strength was assessed manually using the standard medical research council (MRC) scale. Sensory involvement was evaluated in terms of level and severity of pain, temperature, vibration and position sense impairment. Charcot–Marie–Tooth Examination Score version 2 (CMTESv2), a subscore of Charcot–Marie–Tooth Neuropathy Score version 2 (CMTNSv2), was used to estimate disease severity and clinical disability in adult patients^28,29^. We selected CMTESv2 rather than CMTNSv2 as recent neurophysiological testing was not available for all patients. Rasch analysis-weighted CMTESv2 (CMTESv2-R) was also calculated and was used to establish disease severity as mild (0–9), moderate (10–18) and severe (≥ 19)^30,31^. We used CMT pediatric scale (CMTPeds) in children aged 3–18^32^. Disability was classified according to CMTPedS scores as mild (0–16), moderate (16–29) or severe (> 29). Intellective functions were assessed with the Wechsler Adult Intelligence Scale for adults and Wechsler Intelligence Scale for Children–Revised for children. Neurophysiological testing was performed with standard techniques. Optic atrophy was assessed by direct fundoscopy and ophthalmologic review and, when deemed necessary, Optical Coherence Tomography (OCT).

### Genetic analysis

Genomic DNA was extracted from peripheral venous blood samples. Sanger sequencing of *MFN2* was performed. If potential pathogenic *MFN2* variants previously undescribed were reported, to exclude other potential genetic causes of the phenotype, patient's DNA was included in a library for the Next-Generation Sequencing (NGS) of a panel of genes causing hereditary neuropathies. NGS was run on an Illumina MiSeq platform, according to the manufacturer instruction. Then, in silico pathogenicity prediction tools were employed, including Combined Annotation Dependent Depletion (CADD), Mutation Taster, Sorting Intolerant From Tolerant (SIFT), Polymorphism Phenotyping version 2 (PolyPhen2), Functional Analysis Through Hidden Markov Models (FATHMM), Mutation Assessor and MutPred2. Candidate variants were screened in Genome Aggregation Database (gnomAD v2.1.1), Exome Aggregation Consortium (ExAC) and 1000 Genomes Project (1000G). Conservation among orthologous genes of the mutated amino acids was analyzed through the National Center for Biotechnology Information (NCBI) HomoloGene. The American College of Medical Genetics and Genomics (ACMG) criteria for interpretation of sequence variants were also used to classify novel variants^33^.

### Protocol approval and informed consent

All patients or their legal representatives signed an informed consent form prior to enrollment. The study was approved by the local Institutional Review Board (Comitato Etico di Milano Area 2 Protocol Number 898_2020bis). The study was performed in accordance with relevant guidelines and regulations.

## Results

### Characterization of patient cohort and genotype–phenotype correlation

We evaluated thirteen patients belonging to ten different families, who showed a clinical and neurophysiological phenotype consistent with CMT2A and carried a pathogenic *MFN2* variant. Demographic and clinical characteristics of patients enrolled are shown in Tables 1 and 2.

**Table 1:** Phenotype and clinical severity of patients with *MFN2* mutations.

Mutation	Patient #	Age at onset	Age at review	Sex	CMTESv2	CMTESv2-R	Ambulation	Motor symptoms	Sensory symptoms
R94Q	01–1	3 y	22 y	M	13	18	Ambulatory (bilateral support, AFOs)	+	+
R104W	02–1	1 y	5 y	M	26 (CMTPeds)	/	Ambulatory (AFOs)	+	−
	03–1	14 y	55 y	M	18	22	Ambulatory (bilateral support)	+	+
	03–2	2 y	23	M	16	20	Non-ambulatory	+	−
	03–3	4 y	20	M	18	24	Non-ambulatory	+	+
T236M	04–1	5 y	8 y	M	12 (CMTPeds)	/	Ambulatory (plantars)	+	−
S249C	05–1	17 y	39 y	F	7	8	Ambulatory (autonomous)	+	−
R280H	06–1	69 y	77 y	F	9	12	Ambulatory (unilateral support)	+	−
	07–1	48 y	76 y	F	14	19	Ambulatory (bilateral support)	+	+
	08–1	58 y	71 y	M	3	3	Ambulatory (autonomous)	+	−
K357E	09–1	1 y	23 y	F	19	26	Non-ambulatory	+	+
A383V	10–1	7 y	48 y	F	12	13	Ambulatory (AFOs)	+	−
	10–2	12 y	58 y	F	11	16	Ambulatory (walking aids)	+	+

*AFO* ankle–foot orthosis, *CMTES* Charcot–Marie–Tooth examination scale, *CMTES-R* Rasch analysis-weighted CMTES, *CMTPeds* CMT Pediatric Scale.

**Table 2:** Clinical features of patients with *MFN2* mutations.

Mutation	Patient #	Proximal weakness UL *	Distal weakness UL *	Proximal weakness LL *	Distal weakness LL *	Cutaneous sensation UL/LL^§^	Pallesthesia UL/LL^§^	Proprioception UL/LL^§^	Optic atrophy	Scoliosis	Intellectual disability	Restrictive lung disease/Non-invasive respiratory support	Additional symptoms
R94Q	01–1	−	+++	+	+++	+/+	−/−	−/−	−	−	−	−	−
R104W	02–1	−	+++	+	+++	−/−	−/−	−/−	+	−	+	−	Bilateral cataracts, epilepsia partialis continua
	03–1	+	+	++	++	−/−	−/+	−/−	+	−	+	−	Lower limbs myoclonus, spastic paraparesis, dysphagia, sensorineural hearing defect
	03–2	+	+++	+++	+++	−/−	−/−	−/−	−	−	+	+	Dysarthria, ataxic gait features
	03–3	++	+++	++	+++	−/+	−/−	−/−	+	−	+	−	Dysarthria, lower limbs myoclonus
T236M	04–1	−	−	+	++	−/−	−/−	−/−	−	−	−	−	−
S249C	05–1	−	+	+	+++	−/−	−/−	−/−	−	−	−	−	−
R280H	06–1	−	+	+	++	−/−	−/−	−/−	+	−	−	−	Sensorineural hearing defect
	07–1	−	++	−	+++	−/−	−/−	−/−	−	−	−	−	Dysphagia, ptosis
	08–1	−	−	−	++	−/−	−/−	−/−	−	−	−	+	MEPs/SSEPs alteration
K357E	09–1	+	+++	+++	+++	+/+	+/+	+/+	+	+	−	+	Vocal cord paresis
A383V	10–1	−	+	+	+++	−/−	−/−	−/−	−	−	−	−	−
	10–2	−	+++	−	+++	−/−	+/+	−/−	−	−	−	−	−

*LL* lower limbs, *LMN* lower motor neuron, *MEP* motor evoked potentials, *SSEP* somato-sensory evoked potentials, *UL* upper limbs.

*Motor weakness assessed by Medical Research Council scale (MRC): UL proximal weakness assessed by deltoids, biceps brachii and triceps, UL distal weakness assessed by first dorsal interosseus, abductor pollicis brevis and adductor digiti minimi muscles, LL proximal weakness assessed by iliopsoas, quadriceps and hamstring muscles, LL distal weakness assessed by anterior tibialis, gastrocnemius and extensor hallucis longus muscles. −: no weakness; +: slight weakness (> / = 4);++: moderate weakness (3 to 4);+++: severe weakness (< / = 3).

^§^Cutaneous sensation is based on pinprick examination: normal is no definite decrease compared to a normal reference point. Pallesthesia is assessed with Rydel-Seiffer tuning fork: normal is ≥ 5. Proprioception is based on joint position sensation. In the Table, cutaneous sensation, pallesthesia and proprioception are defined as normal (+) or impaired (−) in upper limbs/lower limbs.

Age of disease onset varied widely, ranging from early childhood to late adulthood (1–69 years, mean 18.5, SD + /− 23.6). Walking difficulties were the most common initial symptoms. Clinical severity also ranged from mild to severe, with most patients presenting a severe phenotype, as already observed by previous studies (Table 1, Supplementary Table 1) ^15,16^. CMTESv2 in adult patients ranged from 3 to 19 points (mean 12.7, SD + /− 5), while Rasch-modified CMTESv2 ranged from 3 to 26 points (mean 16.5, SD + /− 6,9). Overall, patients with childhood-onset CMT2A (1–20 years of age) presented a more severe phenotype comparing with patients with adult-onset CMT2A (> 20 years of age). Interestingly, all the three adult-onset CMT2A patients carried the p.R280H mutation.

Clinical sensory involvement was reported only in 6/13 (46%) patients and did not seem to be associated to specific mutations nor to age of onset. Additional symptoms were described in a total of 8/13 (61%) patients. Among them, optic atrophy and reduced visual acuity occurred in 5/13 (38%) patients, in line with previous reports^18^.Furthermore, one p.R280H carrier (08-1) showed concomitant alterations in motor and somatosensory evoked potentials (MEP/SSEP). Two patients, one carrying the p.R104W mutation (03-1) and one carrying the p.R280H mutation (06-1), presented a sensorineural hearing defect. Another carrier of the p.R280H mutation (07–1) displayed dysphagia and ptosis.

As already reported, all subjects carrying the p.R104W variant presented central nervous system involvement with intellectual disability and developmental delay, in some cases with dysarthric speech (patients 03-2 and 03-3), spastic paraparesis (patient 03-1) and ataxic gait (patient 03-2). In addition to that, three patients among them had epileptic manifestations, namely lower limbs myoclonus in two patients from the same family (03-2, 03-3) and epilepsia partialis continua in a third, unrelated patient (02-1). Two patients (02-1, 03-1) displayed punctate white matter alterations at MRI investigation.

### Characterization of MFN2 genetic spectrum and identification of one novel mutation

Except for the proband (09-1), all patients carried a known heterozygous missense *MFN2* mutation already associated to CMT2A phenotype (p.R94Q^5,14,15,18,23,34,35^, p.R104W^16,22^, p.T236M^34^, p.S249C^16^, p.R280H^5,14,16–18,23^, p.A383V^16,36,37^). Most of them (p.R94Q, p.R104W, p.T236M, p.S249C and p.R280H) are located within the highly conserved GTPase domain (Fig. 1A). The affected members of three separate kindreds carried the p.R280H mutation. The p.R104W mutation was observed in four patients from two families. The other mutations (p.R94Q, p.T236M, p.S249C, p.A383V) were reported in one family each.

We detected one novel, heterozygous pathogenic variant, namely the c.1069A > G, p.K357E (NM_014874). The mutation, located in *MFN2* exon 11, is absent from public databases and is predicted as pathogenic by in silico tools (Supplementary Table 2). *MFN2* sequencing in the parents excluded the presence of the variant, thus confirming its de novo occurrence (Fig. 1C). The p.K357E replaces a highly conserved lysine (Fig. 1B) in the R3 region (Fig. 1A), which was found to be necessary for mitochondrial fragmentation and fusion in vitro^38^. A different amino acid substitution at the same site (c.1071G > C, p.K357N) was previously described by Kijima et al. in 2005^34^. The variant fulfils the ACMG criteria for a likely pathogenic variant^33^.

The proband carrying the p.K357E mutation presented a severe, early-onset phenotype characterized by severe weakness and muscle atrophy in both proximal and distal segments of lower limbs and in the distal segments of the upper limbs, and mild weakness and atrophy in the proximal segments of the upper limbs. The patient is not able to walk autonomously, uses the wheelchair and needs assistance for daily activities and self-care. She had scoliosis and foot deformities (*pes cavus)*, and a severe restrictive lung disease with respiratory insufficiency requiring nocturnal non-invasive ventilatory support (NIV). She also displayed complete loss of tactile, vibratory and proprioceptive sensation, optic atrophy and bilateral vocal cord paresis determining dysphonia.

**Figure 2:**
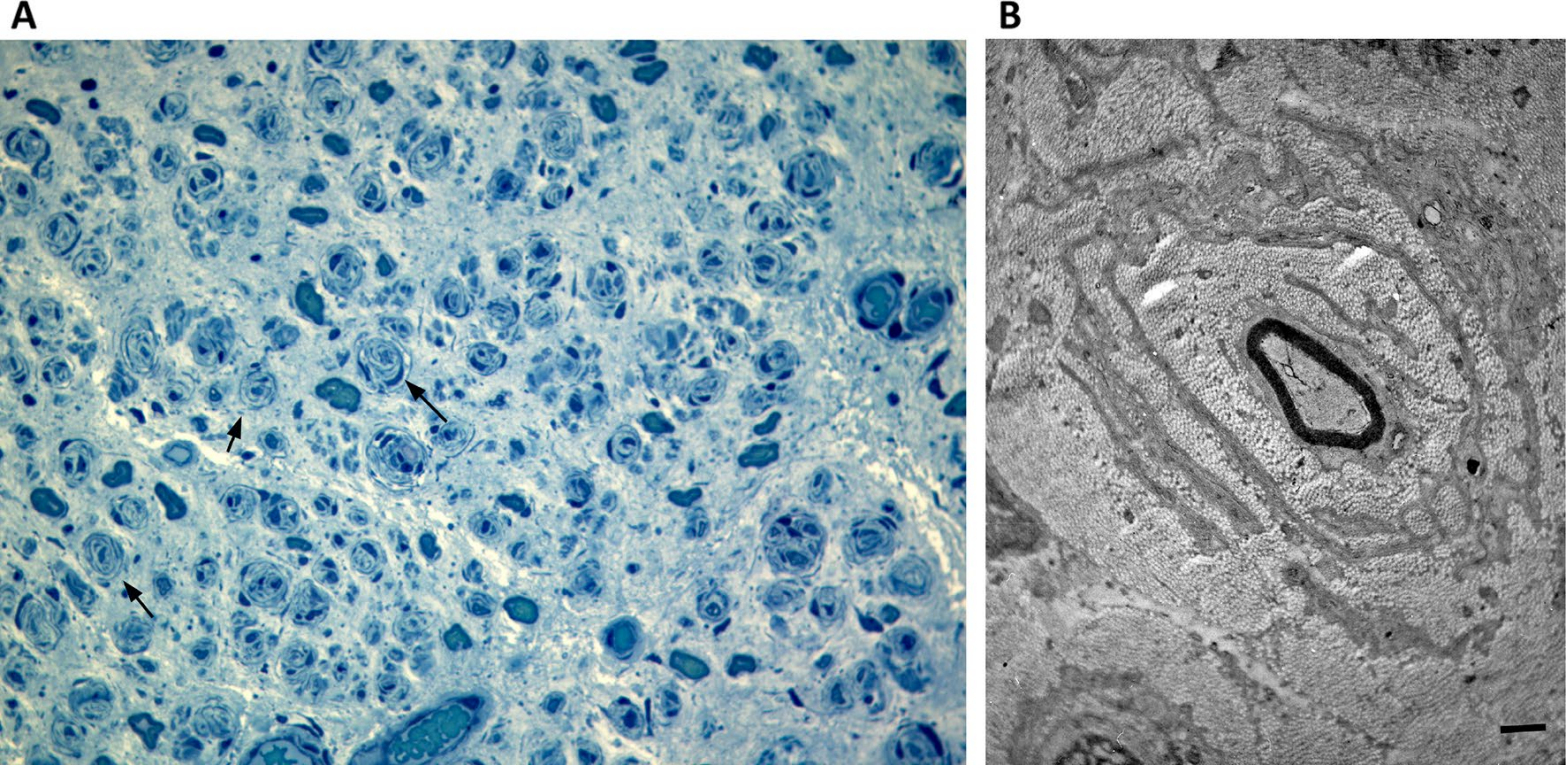
Nerve biopsy of patient carrying the p.K357E variant. (**A**) Sural nerve, semithin section, toluidine blue (400× magnification): several onion bulbs (arrows). (**B**) Electron micrograph; transverse section. One myelinated axon is surrounded by concentric proliferation of Schwann cells. Bar = 1 µm.

The biopsy of the sural nerve was consistent with the clinical severity of the disease showing an almost complete absence of fibers, important connective substitution and several onion bulb formations (Fig. 2), as reported in severe CMT2A cases ^22,39,40^.

## Discussion

We identified seven pathogenic *MFN2* mutations (p.R94Q, p.R104W, p.T236M, p.S249C, p.R280H, p.A383V, p.K357E), which co-segregate with CMT2A phenotype in thirteen patients of ten separate families, including the novel heterozygous p.K357E. We observed the prevalence of moderate or severe phenotypes (38% respectively) among our cohort. The mean CMTESv2 score at review was 12.7, similarly to what has been observed by Pipis and colleagues in a large natural history study^16^, and a great proportion of patients (11, 85%) was not able to walk independently but required aids or wheelchair. Despite the limited number of patients in our cohort and the variable age at review do not allow to draw definite conclusions, these data demonstrate the presence of moderate-to-severe burden of disease in daily life, as opposed to other forms of CMT^41–43^.

As regards genotype–phenotype correlation, the small number of patients does not allow to make precise inferences. It is interesting to note that the p.R280H mutation caused a late-onset phenotype in all the three carriers. This mutation has been previously associated with a highly variable age at onset^16–18,44^. Some of our patients had pure motor neuropathies whereas others showed symptoms and signs of large sensory fibers involvement. These discrepancies were not mutation specific as some variants (p.R104W, p.R280H, p.A383V) were associated with either motor or sensorimotor phenotypes in different individuals. Proximal weakness of lower limbs appeared quite frequently in our patients, suggesting that CMT2A impairment might be less length dependent than other forms of CMT, as already hypothesized by other authors^15,23^. However, proximal involvement in some of our patients may be as well due to the presence of a severe length dependent axonal neuropathy, typical of CMT2A. Therefore, the hypothesis of a non-length dependent proximal involvement remains purely speculative. Notably, three probands carrying the p.R104W mutation suffered from epileptic manifestations, namely myoclonus in two probands from the same kindred and epilepsia partialis continua in a third, unrelated proband. Even though this variant is known to cause an encephalopathic syndrome with developmental delay^16^, epileptic phenomena have been reported only sporadically, in the form of temporo-parietal slow waves^45^ or complex partial seizures with abdominal aura^46^. Thus, the epileptic symptoms observed in our patients further expand the spectrum of manifestations known to occur in the carriers of the R104W variant. Proband 09-1, carrier of the p.K357E mutation, displays a severe phenotype, with severe motor and sensory involvement in both upper and lower limbs, respiratory insufficiency, skeletal deformities, optic atrophy and bilateral vocal cord paresis.

Most of the mutations detected in our series are located in the MFN2 dynamin-GTPase domain, as reported in other studies done so far^15,16^. Nevertheless, the muted amino acid of the novel p.K357E mutation is located outside this functional domain, in a helix bundle formed by helices from the N-terminal and the C-terminal parts of the MFN2 protein (highly conserved R3 region). This helix bundle is required for the expression of the GTPase domain. Furthermore, its structural integrity allows close contact between membranes from adjacent mitochondria, thus resulting in correct mitochondrial fusion^38^. Notably, the impairment in mitochondrial fusion has been proposed as one of the pathogenic mechanisms linking MFN2 dysfunction to neuronal die-back and degeneration^47^. The recent finding that MFN2 agonists reverse mitochondrial defects in CMT2A preclinical models, by mimicking the peptide-peptide interface of MFN2 and consequently activating MFN2 and promoting mitochondrial fusion, further confirms this observation^25,48^. Intriguingly, a report by Kijima and colleagues described a patient with a different amino acid substitution at the same site (p.K357N), presenting with mild-to-moderate symptoms and onset in early childhood. The phenotype severity differed markedly from that of our proband, who harbors the p.K357E mutation. Although we cannot be sure of the reason of such differences in genotype–phenotype correlation, it may be that the substitution of a lysine (K), which is positively charged, with an asparagine (N), a polar amino acid, does not alter the structure and the ionic charge of the helix bundle as the substitution with a glutamic acid (E), which is negatively charged. The specific amino acid might influence the interaction between mitochondrial membranes and, therefore, have a different impact on the regulation of mitochondrial fusion.

In conclusion, our work expands the genetic spectrum of CMT2A by providing a detailed description of clinical features of CMT2A patients who underwent genetic analysis and were confirmed to carry either a known or a novel pathogenic mutation of *MFN2*.

## Supplementary Information

Supplementary Information.

## Data Availability

The data that support the findings of this study are available on request from the corresponding author. The data are not publicly available due to privacy or ethical restrictions.

## Abbreviations

CMT: Charcot–Marie–Tooth
CMTESv2: Charcot–Marie–Tooth Examination Score version 2
CMTESv2-R: Rasch analysis-weighted CMTESv2
CMTNSv2: Charcot–Marie–Tooth Neuropathy Score version 2
CMTPeds: CMT pediatric scale
MFN1/2: Mitofusin1/2
MNCV: Motor nerve conduction velocity
NIV: Non-invasive ventilatory support
