# Supplementary material for: Clinical and genetic features of a cohort of patients with MFN2-related neuropathy
Source: Sci Rep. 2022 Apr 13;12:6181. doi: 10.1038/s41598-022-10220-0 (PMC9008012; doi:10.1038/s41598-022-10220-0)
Supplement: Supplementary file 1 — Supplementary Information. [file 41598_2022_10220_MOESM1_ESM.docx]

**Supplementary Material**

**Supplementary Table 1. Clinical and demographic features of patient cohort**

| **Age, y, mean +/- SD (range)** | 40,4 +/- 25,4 (5-77) |
| --- | --- |
| **M:F** | 7:6 |
| **Age at onset**  **y, mean +/- SD (range)** | 18,5 +/- 23,6 (1-69) |
| **CMTES, mean +/- SD (range)** | 12,7 +/- 5 (3-19) |
| **CMTES-R, mean +/- SD (range)** | 16,5 +/- 6,9 (3-26) |
| **Motor symptoms, n. (%)** | 13 (100%) |
| **Sensory symptoms, n. (%)** | 6 (46%) |
| **Optic atrophy, n. (%)** | 5 (38%) |
| **Mild phenotype, n. (%)** | 3 (23%) |
| **Moderate phenotype, n. (%)** | 5 (38%) |
| **Severe phenotype, n. (%)** | 5 (38%) |

**Supplementary Table 2** – *In silico* pathogenicity prediction. Assessment of the deleterious impact of the *MFN2* p.K357E variant by *in silico* prediction tools CADD, Mutation Taster, SIFT, PolyPhen2, FATHMM, Mutation Assessor, and MutPred2.

| *MFN2* – c.1069A>G – p.K357E | |
| --- | --- |
| **CADD** | Deleterious  (28.7) |
| **Mutation Taster** | Disease causing  (0.99) |
| **SIFT** | Damaging  (0.000) |
| **PolyPhen2** | Probably damaging  (0.99) |
| **FATHMM** | Damaging  (-4.35) |
| **Mutation Assessor** | Medium functional impact  (3.245) |
| **MutPred2** | Pathogenic  (0.742) |
